# BRAF^V600E^ and TERT promoter C228T mutations on ThyroSeq v3 analysis of delayed skin metastasis from papillary thyroid cancer: a case report and literature review

**DOI:** 10.1186/s12957-023-02937-7

**Published:** 2023-02-17

**Authors:** Jee-Hye Choi, Hyeong Won Yu, Ja Kyung Lee, Woochul Kim, June Young Choi, Hee Young Na, So Yeon Park, Chang Ho Ahn, Jae Hoon Moon, Sang Il Choi, Ho-Young Lee, Won Woo Lee, Wonjae Cha, Woo-Jin Jeong

**Affiliations:** 1 Department of Surgery, Seoul National University Bundang Hospital and College of Medicine, 82, Gumi-Ro 173 Beon-Gil, Bundang-GuGyeonggi-Do, Seongnam-Si, 13620 Korea; 2 Department of Surgery, The Mount Sinai Hospital, Icahn School of Medicine at Mount Sinai, 1468 Madison Ave, New York, NY 10029 USA; 3 Department of Pathology, Seoul National University Bundang Hospital and College of Medicine, 82, Gumi-Ro 173 Beon-Gil, Bundang-Gu, Seongnam-Si, Gyeonggi-Do, Korea; 4 Department of Internal Medicine, Seoul National University Bundang Hospital and College of Medicine, 82, Gumi-Ro 173 Beon-Gil, Bundang-Gu, Seongnam-Si, Gyeonggi-Do, Korea; 5 Department of Radiology, Seoul National University Bundang Hospital and College of Medicine, 82, Gumi-Ro 173 Beon-Gil, Bundang-Gu, Seongnam-Si, Gyeonggi-Do, Korea; 6 Department of Nuclear Medicine, Seoul National University Bundang Hospital and College of Medicine, 82, Gumi-Ro 173 Beon-Gil, Bundang-Gu, Seongnam-Si, Gyeonggi-Do, Korea; 7 Department of Otorhinolaryngology-Head and Neck Surgery, Seoul National University Bundang Hospital and College of Medicine, 82, Gumi-Ro 173 Beon-Gil, Bundang-Gu, Seongnam-Si, Gyeonggi-Do, Korea

**Keywords:** Papillary thyroid cancer, Metastatic papillary thyroid cancer, Skin metastasis, ThyroSeq

## Abstract

**Background:**

Skin metastasis from papillary thyroid cancer (PTC) is a rare entity that can occur up to decades after treatment of the primary tumor. Here, we present a patient who developed skin metastasis 10 years after treatment of her primary tumor and describe the molecular findings of the metastatic lesion.

**Case presentation:**

A 44-year-old female with a history of PTC who underwent a total thyroidectomy and radioactive iodine (RAI) treatment 10 years ago presented with a 1.3-cm skin lesion along the prior thyroidectomy scar. A biopsy revealed metastatic PTC, and the patient underwent surgical excision of the lesion. ThyroSeq molecular testing showed the copresence of BRAF^V600E^ mutation and TERT promoter C228T mutation. The patient subsequently received one round of adjuvant RAI therapy.

**Conclusions:**

A high index of suspicion is warranted in patients with a history of PTC who develop a skin lesion, even several years after remission of the primary disease. In patients with high-risk mutations, such as BRAF^V600E^ and TERT promoter C228T mutations, long-term surveillance of disease recurrence is particularly important.

## Background

Papillary thyroid cancer (PTC) is associated with an excellent prognosis, with a 10-year survival rate above 90% [1]. Consistent with the indolent nature of the disease, distant metastasis from PTC is uncommon and is found in only 1–4% of cases. However, when present, it is associated with a poor prognosis, lowering the 5-year survival to 28–53.3% [2, 3]. The most common sites of distant metastasis are the lungs and bones, with scant reports of more unusual sites such as the liver, brain, kidney, pancreas, adrenal glands, and skin [2, 4].

Skin metastasis from PTC is extremely rare, occurring in less than 1 in 1000 patients with PTC [5, 6]. These cases are reported mainly through case reports and demonstrate a variable presentation in terms of location of the metastatic lesion, disease state, and the timing of discovery in relation to the initial diagnosis of PTC. The onset of skin metastasis ranges from within 1 year to as late as 30 years after the initial diagnosis of PTC, with the median onset of 8.25 years from the initial treatment [7].

In this report, we present a case of skin metastasis that developed 10 years after the initial treatment of PTC. In addition to describing the clinical presentation and pathology findings as published in preexisting literature, we also performed genetic and molecular analyses of the metastatic tumor using the ThyroSeq® Genomic Classifier test to further investigate this rare disease entity.

## Case presentation

A 44-year-old woman was found to have a small erythematous skin lesion on the right side of the neck at a routine annual follow-up with an endocrinologist. The patient had a history of PTC diagnosed 10 years ago and was treated with a total thyroidectomy and 120 mCi of adjuvant radioactive iodine (RAI) at an outside hospital. Since her treatment, she had been taking 150 mcg of levothyroxine daily and continued yearly follow-up with her endocrinologist. Prior to the most recent endocrinology visit that revealed the new skin lesion, the patient had no other documented evidence of disease recurrence, and when interviewed further, the patient did not recall when the skin lesion first appeared.

**Fig. 1:**
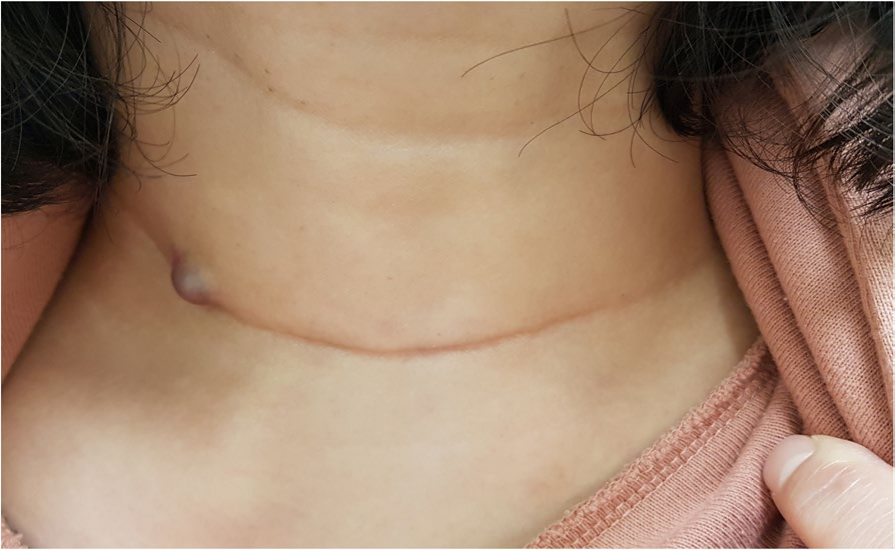
A small 1.3-cm skin lesion found on the right side of the prior thyroidectomy scar

**Fig. 2:**
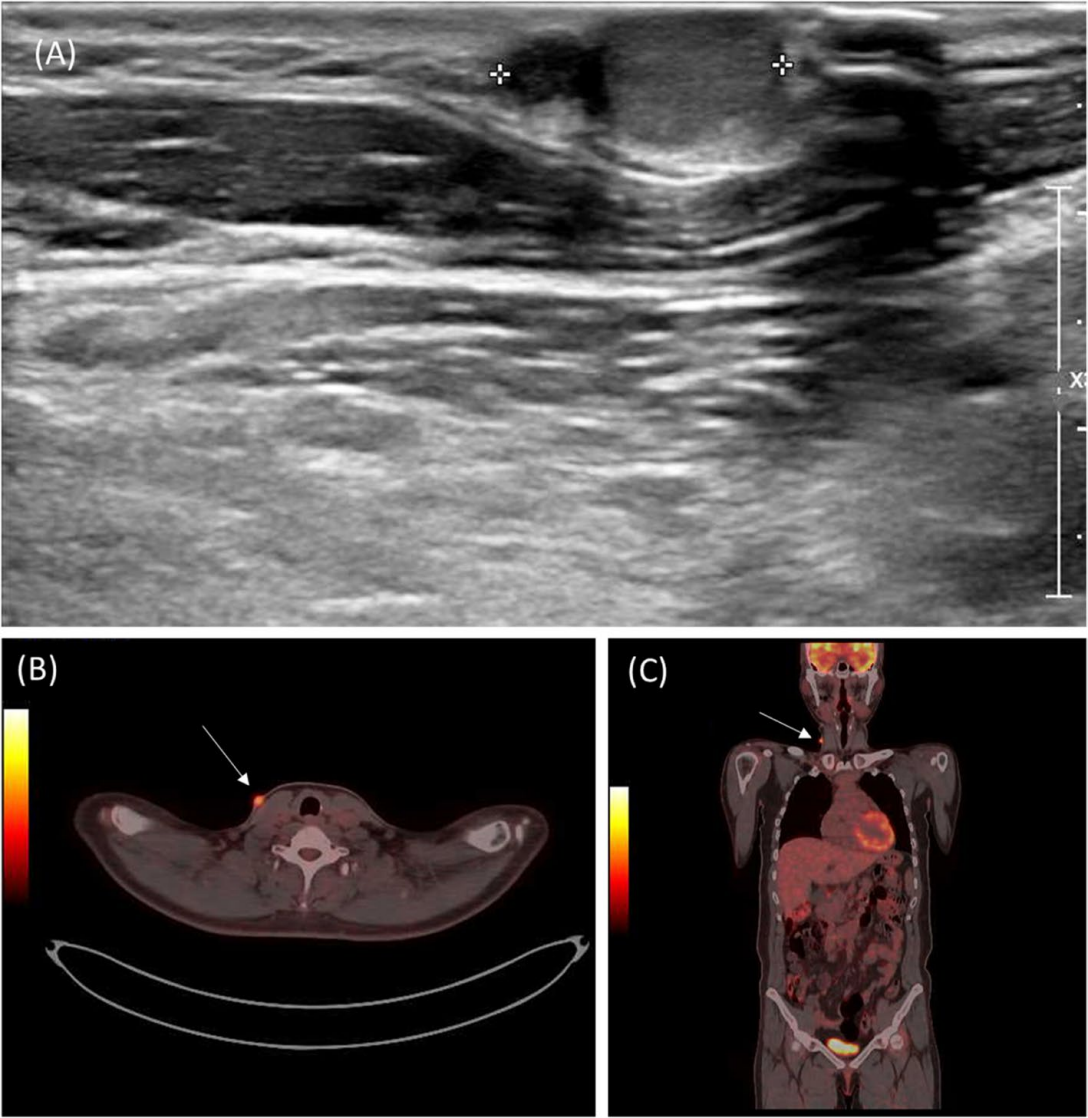
**A** Preoperative ultrasound of the right neck skin lesion. **B** and **C** PET/CT showed a hypermetabolic lesion in the right neck and no other abnormal hypermetabolic lesions

Physical exam revealed a 1.3-cm skin lesion at the edge of the prior thyroidectomy scar (Fig. 1). Ultrasound of the neck confirmed a 1.3-cm subcutaneous nodule, consistent with the physical exam finding (Fig. 2). Several reactive lymph nodes were also detected at levels 1 and 2 of the right neck, but there was no evidence of local recurrence in the operative bed. Labs were unremarkable with the following results: thyroid-stimulating hormone (TSH), 0.05 uIU/ml; free T3, 2.79 pg/ml; free T4, 1.69 ng/dl; thyroglobulin, 1.00 ng/ml; and anti-thyroglobulin antibody, < 20 U/ml. Differential diagnosis at the time included intradermal nevus, hemangioma, and metastasis from prior thyroid cancer. The patient was referred to dermatology for biopsy of the lesion.

Punch biopsy was performed in two separate sites: the center of the lesion and the periphery of the lesion. The biopsy of the center revealed carcinoma in dermis with a papillary growth pattern and intranuclear pseudoinclusions, consistent with metastatic papillary thyroid carcinoma. The peripheral biopsy showed mild superficial perivascular lymphocytic infiltration. Whole-body PET/CT was then taken (Fig. 2), which showed a focal hypermetabolic skin lesion in the right lower neck, consistent with the known cutaneous metastasis; no other abnormal hypermetabolic lesions were found. Based on these findings, the patient was diagnosed with an isolated metastatic site in the skin and was referred to surgery.

**Fig. 3:**
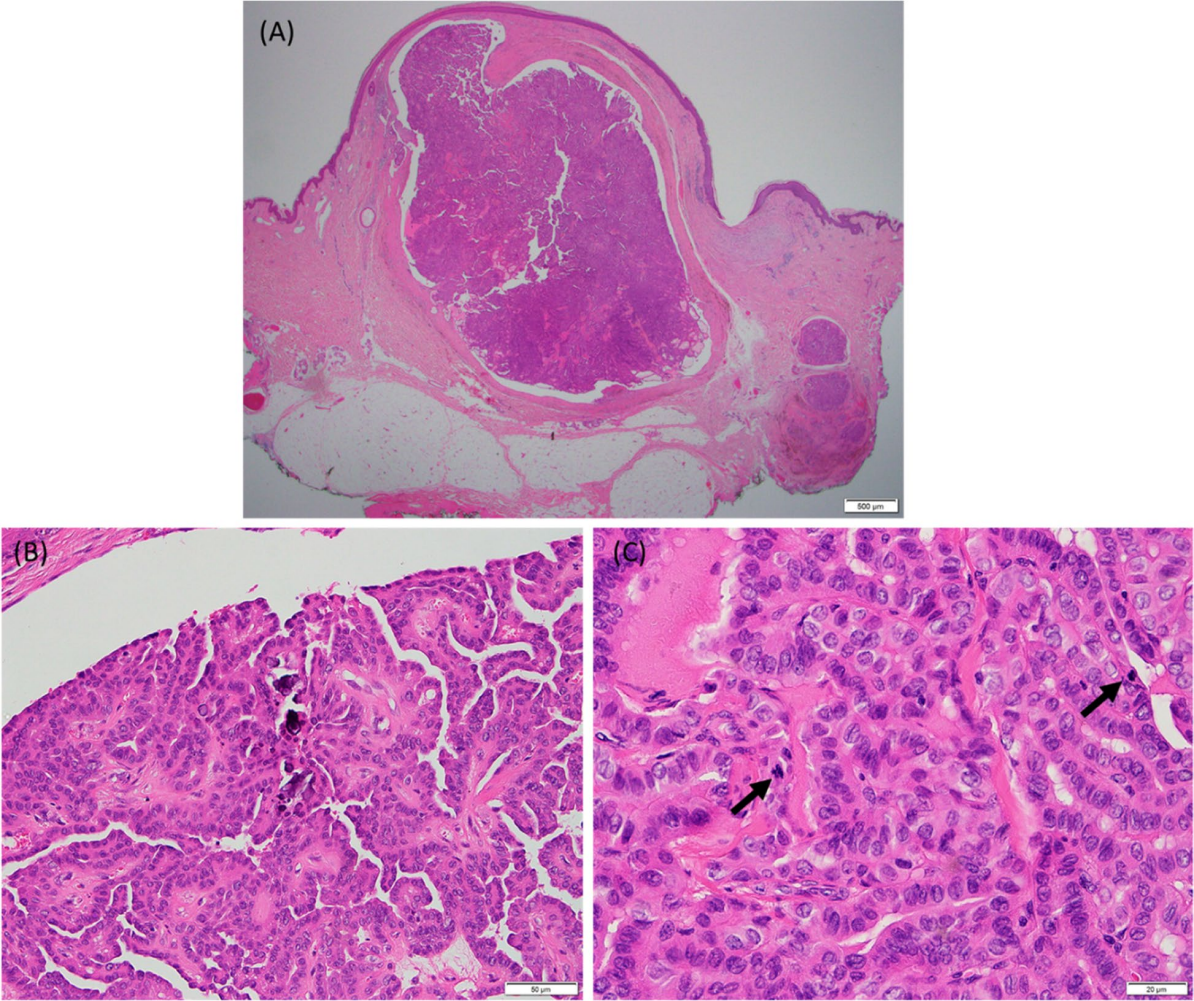
**A** Scan view showing a metastatic papillary thyroid carcinoma in the dermis. Scale bar, 500 µm. **B** Complex papillae with psammomatous calcification was observed (× 200). Scale bar, 50 µm. **C** Increased mitoses (up to 3/10 HPF) were noted in the high-power field (× 400). Scale bar, 20 µm

The patient underwent surgical excision of the metastatic lesion with an elliptical incision. A small area of underlying subcutaneous fat showed nodularity and was removed as well. Surgical pathology revealed three separate foci of metastatic PTC measuring 1.0 × 0.8 × 0.7 cm, 0.3 × 0.2 cm, and 0.2 × 0.2 cm. The subcutaneous fat harbored no metastasis, and the resection margin was clear (Fig. 3). The patient recovered appropriately postoperatively and was discharged home.

**Fig. 4:**
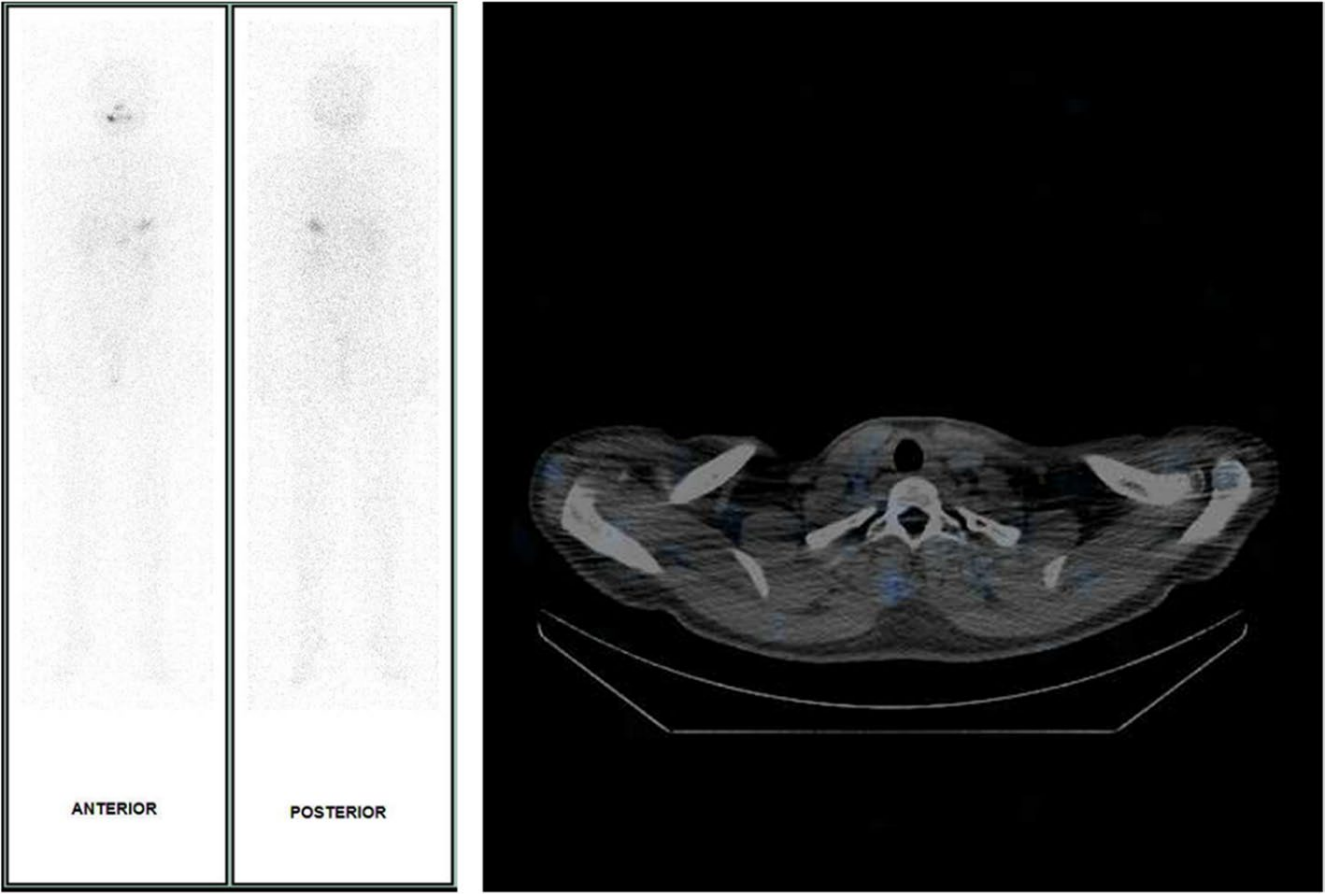
Radioactive iodine scan revealed no remnant thyroid activity and no other abnormal increased iodine uptake, including in the right neck skin area

Genetic and molecular analysis of the metastatic tumor was performed using the ThyroSeq® v3 Genomic Classifier test, and the results showed both BRAF^V600E^ and TERT^C228T^ mutations. Based on the high-risk profile, the patient was referred to nuclear medicine and received one round of adjuvant RAI therapy with a dose of 100 mCi (Fig. 4).

## Discussion and conclusions

Skin metastasis from PTC has been described in prior case reports, but the incidence remains extremely low. Most skin metastases are located in the scalp, face, and neck, which is likely due to the rich lymphatic and vascular supply in these areas [5–9]. Other locations such as the medial arm and supraclavicular fossa have also been described [6, 10].

Similar to most distant metastases, the potential mechanisms underlying skin metastasis include direct extension and hematogenous or lymphatic spread of the primary tumor [7, 8]. Additionally, there have been reports of metastatic skin lesions away from the thyroidectomy scar but along the prior core needle biopsy tract, suggesting needle-tract seeding as another possible route of metastasis [11, 12]. Another unusual mechanism of skin metastasis that had been suggested involves the use of surgical drain, with one report describing a patient who developed a metastatic skin lesion from PTC along the prior site of surgical drain placement [13]. In the present case, the skin metastasis was located on the edge of the prior thyroidectomy scar, which suggests several possible explanations including needle-tract seeding, drain-site seeding, hematogenous or lymphatic spread, or seeding of tumor cells during the index surgery. However, because the index surgery occurred 10 years ago at an outside hospital, details of the initial work-up and the operation—including the exact tract of the prior needle biopsy, use of a surgical drain tube and its location, or skin contamination during thyroid extraction—could not be obtained to suggest one route as a more likely explanation over another.

The skin lesion itself may manifest as a flesh-colored or pigmented papule, plaque, or nodule with or without ulceration [7, 8]. Because its appearance can vary and resemble other skin lesions, diagnosis can be challenging without a high suspicion, especially since these metastases can occur several years after remission of the primary cancer. The present case also demonstrates that skin metastasis can occur even without marked elevation of the thyroglobulin level. At our institution, we typically suspect a tumor recurrence in TSH-suppressed patients when their thyroglobulin levels increase above 2.0 ng/mL. In the present case, the patient was found to have a thyroglobulin level of 1.0 ng/mL, a level that is higher than expected in setting of TSH suppression and prior total thyroidectomy, but not significantly elevated enough to suggest a recurrence. Therefore, a high index of suspicion is warranted for any patient with a prior history of PTC presenting with a skin lesion—even when the work-up shows a low or borderline thyroglobulin level—because such lesion can still represent a metastatic or recurrent disease and can appear several years after the initial remission of the primary tumor.

Because the condition is rare, there are no standardized guidelines regarding treatment. The treatment usually involves excision of the metastatic site, and additional therapies including RAI, external-beam radiotherapy, or targeted therapy such as sorafenib or vandetanib may be used [6, 7]. The utility of adjuvant therapy depends on several factors including the iodine avidity of the tumor, the extent of residual disease, and the molecular characteristics of the lesion. Because PTC with skin metastasis is often associated with disseminated disease with a reported mean survival time of 19 months [5], a thorough metastatic work-up and patient counseling on prognosis should precede any therapeutic decisions.

The molecular characteristics of thyroid cancer have long been used to both predict the prognosis of the disease and to determine any mutations that may be subject to targeted therapy; however, few studies have reported the molecular features of PTC with skin metastasis. A study published by Erickson et al. in 2007 investigated the BRAF gene in 11 patients with PTC with skin metastases and found that 5/11 patients had a BRAF mutation [14]. A case report by Cohen et al. described a patient with PTC metastatic to skin, which was found to have a rearranged during transfection (RET) receptor tyrosine kinase mutation. The patient was treated with vandetanib, a RET inhibitor, and survived for at least 1.5 years after initiation of the drug treatment [6]. While such attempts have been made to discover mutations and apply targeted therapy for treatment of PTC with skin metastasis, further investigations are needed to better understand the composition and significance of molecular characteristics in management and prognosis of the disease. In the present case, we sought to gain a deeper understanding of the genetic profile of this rare form of metastasis by performing the ThyroSeq® v3 Genomic Classifier test. To the best of our knowledge, this is the first reported case of skin metastasis from PTC for which a comprehensive molecular analysis was performed using ThyroSeq.

The test identified both BRAF^V600E^ and TERT promoter C228T mutations. BRAF mutation is well-known to be the most frequently mutated gene in PTC, with a higher frequency in the Asian population with a reported prevalence of 68.7% among PTCs; however, TERT promoter mutation, which is less common in general, is even rarer in the Asian population, accounting for only 6.8% of all PTCs [15]. Therefore, coexistence of both mutations was an unexpected finding. Although BRAF mutation had been associated with lymph node metastases, extrathyroidal extension, tumor size, and advanced disease stage [16], more recent studies have questioned its impact on risk stratification and prognosis for PTC [17]. However, TERT promoter mutation has been found to be an independent predictor of distant metastasis, recurrence, and disease-free survival [18–21]. Moreover, its copresence with BRAF mutation has been associated with even more aggressive clinicopathological characteristics [22]. Therefore, the presence of TERT mutation in the present case, especially in copresence with BRAF mutation, is consistent with the aggressive nature of the patient’s disease with its recurrence in a rare metastatic site several years after the initial treatment. Unfortunately, the primary tumor from 10 years ago was not available for ThyroSeq analysis, and it is unclear whether the two mutations were present in the primary tumor or the mutations occurred later in the course of the disease leading to a late presentation of the metastasis.

The present case demonstrates that PTCs that appear to be in remission for up to 10 years can still develop a recurrence manifested as skin metastasis. Other reports have demonstrated that these metastases can occur even 30 years after the initial treatment [7], and a high index of suspicion is important for any patient with a history of PTC who develops an upper body skin lesion. The present case also suggests that patients with high-risk mutations—such as copresence of BRAF and TERT promoter mutations, as in our case—in the primary tumor can especially benefit from long-term surveillance of recurrence, as their disease is associated with more aggressive characteristics and distant metastases.

## Data Availability

Data sharing is not applicable to this article as no datasets were generated or analyzed during the current study.

## Abbreviations

PTC: Papillary thyroid cancer
RAI: Radioactive iodine
TSH: Thyroid-stimulating hormone
T3: Triiodothyronine
T4: Thyroxine
PET: Positron emission tomography
CT: Computed tomography
